# A Salutogenic Perspective on End-of-Life Care among the Indigenous Sámi of Northern Fennoscandia

**DOI:** 10.3390/healthcare9060766

**Published:** 2021-06-19

**Authors:** Lena Kroik, Carol Tishelman, Krister Stoor, Anette Edin-Liljegren

**Affiliations:** 1 Department of Nursing, Umeå University, 901 87 Umeå, Sweden; anette.liljegren@regionvasterbotten.se; 2 The Center for Rural Medicine, Region Västerbotten, 923 31 Storuman, Sweden; 3 LIME/Division of Innovative Care Research, Karolinska Institutet, 171 77 Stockholm, Sweden; carol.tishelman@ki.se; 4 Stockholm Health Care Services, 171 77 Stockholm, Sweden; 5 Department of Language Studies, Umeå University, 901 87 Umeå, Sweden; krister.stoor@umu.se; 6 Centre for Sámi Studies-Várdduo, Umeå University, 907 87 Umeå, Sweden

**Keywords:** indigenous research, salutogenesis, end-of-life, ethnic groups, Sámi, Sweden, sense of community coherence

## Abstract

There is limited empirical data about both health and end-of-life (EoL) issues among the Indigenous Sámi of Fennoscandia. We therefore aimed to investigate experiences of EoL care and support among the Sámi, both from the Sámi community itself as well as from more formalized health and social care services in Sweden. Our primary data source is from focus group discussions (FGDs) held at a Sámi event in 2017 with 24 people, complemented with analysis of previously collected data from 15 individual interviews with both Sámi and non-Sámi informants familiar with dying, death and bereavement among Sámi; “go-along” discussions with 12 Sámi, and individual interviews with 31 Sámi about advance care planning. After initial framework analysis, we applied a salutogenic model for interpretation, focusing on a sense of community coherence. We found a range of generalized resistance resources in relation to the Sámi community, which appeared to support EoL care situations, i.e., Social Organization; Familiarity with EoL Care, Collective Cultural Heritage; Expressions of Spirituality; Support from Majority Care Systems; and Brokerage. These positive features appear to support key components of a sense of community coherence, i.e., comprehensibility, meaningfulness and manageability. We also found relatively few, but notable deficits that may diminish the sense of community coherence, i.e., lack of communication in one’s own language; orientation, familiarity and/or agreement in contacts with formal health and social care systems; and/or support from extended family. The results suggest that there is a robust basis among Sámi for well-functioning EoL care; a challenge is in developing supportive interactions with the majority health and social care systems that support and complement these structures, for partnership in developing care that is meaningful, comprehensible and manageable even in potentially difficult EoL situations.

## 1. Introduction and Aim

This article derives from a larger program of research, DöBra (DöBra is a pun in Swedish, literally meaning “dying well” and figuratively used as “awesome”), based on a public health approach to end-of-life (EoL) care. DöBra is composed of several specific projects which share the general goal of aiming to promote constructive change to support a better quality of life and death among the general population, in specific subgroups, and interventions directed to professional groups caring for dying individuals, their friends and families. These projects also share some methodological features, working with innovative approaches in partnership with different publics and disciplines. Prompted by the limited empirical data about both health and EoL issues, the article presented here is one of several in a project focusing on EoL issues among the Indigenous Sámi people in Sweden [1,2,3]. While there is increasing international attention paid to Indigenous health issues, most EoL-related research still derives from Australia, New Zealand and Canada [4,5]. 

The Sámi have their origin in Sápmi, an area in Northern Fennoscandia. In Sweden today the Sámi population is estimated at between 17,000–40,000 people [6,7]; lack of clarity is a result of ethnic background not being explicitly documented in the country. About 70% of Sámi live in the rural and remote northern half of Sweden [8], with over 4600 involved in reindeer husbandry to some extent [9]. This involves “mobile pastoralism” [10], as the owners follow the reindeer in seasonal wandering across extensive areas. This way of life does not always mesh with the organization of Swedish society [11]. 

Particular regional and municipal areas in which many Sámi live have a formal mandate to support Sámi opportunities to develop and preserve their culture [12]. This means that government agencies in these areas must actively promote the maintenance and continued development of the Sámi identity, including the Sámi language. In theory, this could include the opportunity for a Sámi person to receive EoL support from health care providers with cultural understanding, knowledge of what it means to live as a Sámi in Sweden today, and with Sámi language skills.

Although limited, the existing research from Sápmi and from other Indigenous contexts gives reason to question the quality of interactions between formal services and Sámi in need of health and social care [13,14]. The overall aim of this article is therefore to investigate experiences of EoL care and support among the Sámi, both from the Sámi community itself as well as from formalized services in the majority society. This includes exploring questions such as who and what is described as providing support in relation to the EoL, what forms of knowledge are referred to, and how the interplay between formal and informal support systems is portrayed.

## 2. Background

A review of palliative care service delivery for Indigenous Peoples’ in Australia, New Zealand, Canada and the U.S. points to commonalities, despite cultural differences, in the importance of cultural aspects, family and network, as well as access to EoL care close to or in the home environment [15,16]. However, this and other research also point to a range of difficulties, often due to living in geographically remote regions, in realizing care provision that incorporates these aspects [17]. 

Recent research from Canada on cancer treatment decisions further highlights the unique health needs of Indigenous peoples, but also how disparities and other difficulties in contact with the health care system are common, with historical origins from colonial times [18]. Difficulties in contact with mainstream care, notably a lack of satisfaction, were also observed with regard to Norwegian Sámi’s contacts with both health and social care systems [19,20]. Numerous factors were found to play a role, i.e., not feeling included in care that was provided without consideration to Sámi language or culture, differences in ways of communicating, particularly around care needs, as well as preconceived notions about Sámi self-sufficiency and reluctance to seek formal care [21]. Blix [22] notes that Sámi may avoid speaking directly about sickness, by using metaphors and other indirect references, a type of indirect communication also addressed by Duggelby et al. [23] in their meta-synthesis of research on Indigenous people’s EoL experiences in English-speaking countries. However, Blix [22] also warns about the risk of over-generalization about indirect communication. 

In her Swedish doctoral thesis, Daerga [24] found that the Swedish health care system does not fit well with the needs of Sámi migrating reindeer herders who are not able to enroll as patients in a particular care facility. These facilities are also often accessible only at times that are not suitable for them. The needs of reindeer husbandry thus were found to lead to delays in care-seeking and therefore a lack of reliable statistics about health issues. Blix [22], while noting few differences in health status and use of care services between the Norwegian Sámi and the rest of the population, also points to a lack of satisfaction with services among the Sámi, but warns about over-generalizations that may result from describing the Sámi as a homogeneous group. 

Even Ness et al. [25] emphasize that the Sámi are not necessarily homogenous even when sharing a specific local background. In their recent Swedish study of older South Sámi people in home care, they found that individuals in this group could have contradictory expectations of nursing care provided by Sámi-speaking staff [25]. 

While relevant, little Sámi-related research, including that named above, focuses on EoL issues. In our previous EoL-specific research [23], we critically examined Kastenbaum’s model of death systems [26] in relation to Swedish Sámi. We found a number of features we call Sámi-relevant, in that they could also be shared by other parts of Swedish society and were often linked to the particular environment in the rural and remote far north. However, we also found Sámi-specific features, closely related to community identity that played a role in relation to dying, death and bereavement. Relationships to nature and seasons linked people, place and time, with the role of the extended family for enculturation of central importance. In contrast to many assumptions, we found that by using a variety of conversational-based data collection and analysis approaches, Sámi people were willing to share their experiences and knowledge related to EoL issues with us. However, the research we conducted previously did not focus on the part of Kastenbaum’s death system related to “care for dying” [1,2,3]; in this article, we make an effort to fill this knowledge gap. 

## 3. Methods

### 3.1. Research Team Investigating Sámi Perspectives on EoL

The research group consists of four people, with both Sámi and non-Sámi backgrounds. Both Sámi researchers, a south Sámi woman PhD student and registered nurse L.K, and K.S, a northern Sámi man with a PhD in Sámi studies, have strong roots in the reindeer-herding community. The non-Sámi researchers are a non-Swedish woman, CT who is a registered nurse PhD living in the capital region of Sweden and a woman PhD, AEL, from northern Sweden, with experience of research involving Sámi health issues. LK, KS, and CT participated in all or part of the data collection underlying this article, along with OL, a non-Sámi RN PhD researcher and project PI until his death in 2018.

### 3.2. Data Collection

The bulk of the data was collected in 2017 at the Sámi Church Days, an event arranged every fourth year by Sámi church organizations in the Nordic countries and Barents region—that is, the whole area considered to be Sápmi, the original home of the Sámi people. This event brings many Sámi together, irrespective of whether they identify as religious or secular, as it is one of few occasions to meet, even across country borders. In 2017, Sweden hosted the Church Days in a small town in Lapland in Northern Sweden. This event focused on Sámi church life, with worship services, talks, lectures and cultural presentations covering a wide range of Sámi-relevant topics. Issues such as Sámi rights, self-determination, sustainable development in the Arctic, reconciliation and further development of Sámi church life were some of the areas discussed. The first author contacted the organizers about their interest in her presenting research findings from the group’s initial studies as a means for both providing feedback to the community and inspiring new group discussion as a form of data collection. Information about the 90-min initiative consisting of both a lecture “When the well of life dwindles-care at the end of life” with subsequent focus-group discussions (FGDs) was disseminated through the program for the Sámi Church Days.

Approximately 50 people attended the lecture, and 24 participated in the following FGDs with all providing informed consent to use the data generated. No pre-registration to this program point was needed. Participants were predominately Sámi with a variety of backgrounds, most from Sweden, but also from Norway and Finland, working with, e.g., reindeer husbandry, in churches, with handcrafts and other forms of Sámi culture, in the health/social care system and as academics and linguists. No demographic data were systematically collected at this event.

Four FGDs with 5–7 participants plus a moderator in each, were held simultaneously in a large room, with each table focusing on a different topic and moderated by one member of the research team. Participants themselves chose which topic they wished to discuss. The four topics were: “Is yoik (a form of Sámi a cappella song, see below) a natural part of dying, death and bereavement (FGD 1, moderator KS)?”; “What role does the landscape/nature play in dying, death and bereavement (FGD 2, LK)?”; “How do the health and social care systems work today for Sámi at the end-of-life (FGD 3, OL)?” and “Who does what in Sámi families at the end-of-life and in its aftermath (FGD 4, CT)?”

The FGDs lasted for about 30 min, with a 15-min joint closing discussion including a summary of the discussion at each table. Each FGD was audio-recorded, as was the joint discussion, and the event photo-documented after receiving permission from all participants.

While the bulk of the data used for analysis was generated as described above, this was complemented with a directed content analysis [27] to find additional relevant data from our combined, previously generated data with 58 other individuals derived from (a) individual interviews (*n* = 15, see [1] for detail) from 2016–2017 with both Sámi and non-Sámi informants with experience of dying, death and bereavement among Sámi; (b) storytelling in go-along group discussions to gravesites at the tree-line with cultural and historical significance for the Indigenous Sámi peoples held in 2017 (*n* = 12 Sámi participants [2]); and (c) brief explorative advance care planning (ACP) discussions with 31 Sámi people with participants at Sámi events in 2019–2020 [3], see Table 1. While the data collection approaches varied, they shared the characteristic of addressing EoL issues in conversational form, and the combined database for this analysis focused on transcript excerpts related to EoL care only.

### 3.3. Ethics

In addition to formal ethical permission granted (2016/02-31, 2016/252-32), we also contacted the Church of Sweden’s research unit and received their permission for data collection at the Sámi Church Days. Prior to ethical review, we composed an information letter and informed consent form for participants. These were professionally translated from Swedish to Norwegian and to the three major Sámi languages.

### 3.4. Data Analysis

**Figure 1:**
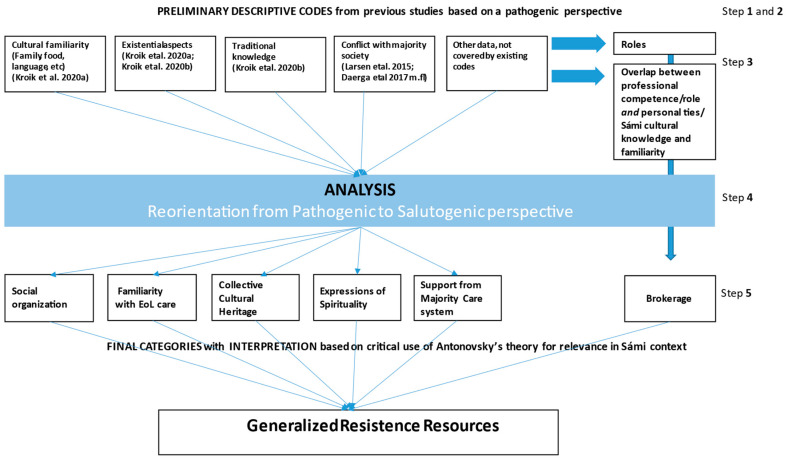
Graphic description of the analytic process.

Analysis initially followed Framework Analysis, focusing on the care of the dying. Framework analysis is an iterative approach [28] that makes use of previous knowledge by questioning its relevance and developing it further in relation to new data, thus heightening its usefulness in practice and policy. The analysis was performed in the 5 steps described by Ward [28]: 1. Familiarization—through immersion in the data, 2. Developing a theoretical framework by identifying recurrent and important themes, 3. Indexing and pilot charting, 4. Summarizing data in analytical framework, and 5. Synthesizing data by mapping and interpreting. These steps are shown in the graphic illustration of the analysis process depicted in Figure 1. 

LK, who led data analysis with the support of the co-authors, began by familiarizing herself with all data, listening to audio recordings and rereading verbatim transcripts repeatedly. In the next stage, she extracted all data relating to the care of the dying up until the time of the funeral and compiled it in a new database. Based on the existing literature, both from this research group’s prior studies and the international literature, preliminary codes were developed and used in initial indexing and pilot charting onto a matrix for each data segment about EoL care. The initial preliminary descriptive codes can be found in Figure 1.

At this point, we began to recognize that the research team, emulating much of the literature, had been expecting a problem-orientation in the data. We instead observed that this focus led us to circular reasoning, without generating new knowledge and did not seem to match the substance of the empirical data. We began to transition from description to analysis and observed several salient features in the data. One feature was that the distinction between professional or formal health and care services versus informal caregiving was not found to be as distinct as expected, resulting in new preliminary codes relating to overlap and integration of formal and informal care systems through the roles people had (Figure 1). The second feature of importance was that much of the data described how difficult situations were dealt with in manners evaluated as positive by the participants. 

This latter feature led to a new stage in the analysis process, illustrated in Figure 1, in which we questioned our own pathogenic assumptions as researchers and made an effort to focus on what new knowledge could be found in these data, which led us to be inspired by Antonovsky’s salutogenic framework [29] for interpretation. This phase was guided by a new question we addressed to the data during analysis: “What is described as leading to positive outcomes in EoL care situations among the Sámi, according to these data?”

As we continued the coding/recoding process, we observed that most of our final categories could be conceptualized according to what Antonovsky described as Generalized Resistance Resources (GRRs) in his early work [30]. GRRs are defined as “any characteristic of the person, the group, or the environment that can facilitate effective tension management” and are described along a continuum with the opposite pole called Generalized Resistance Deficits (GRDs) when such resources are lacking (p. 28). Antonovsky means that GRRs are important in that they provide life experiences that promote the development and maintenance of what he calls a strong Sense of Coherence (SOC), whereas GRDs provide experiences that wear away at a SOC (p. 129).

While a SOC is generally discussed in relation to individuals, several researchers also argue its relevance in more collective situations, e.g., families and communities [31]. According to Peled, Sagy and Braun-Lewensohn [32], a sense of community coherence is based on the same three key components as individual SOC. In this context, they define *comprehensibility* as the “sense of predictability and security felt by the members of a community and the extent of which the community is comprehensible”; *manageability* as “the ability of the community to assist its members in times of need”; and *meaningfulness*, the motivational component, as “the ability of the members of the community to express themselves in order to feel a higher level of satisfaction and interest within the community”. 

**Figure 2:**
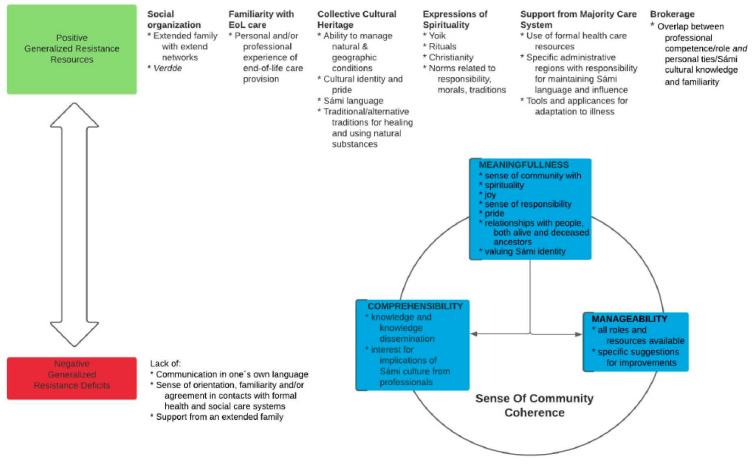
Graphic representation of a description and interpretation of the results.

The final interpretative phase illustrated in Figure 1, involved again perusing all data in relation to GRRs, GRDs and the components of a SOC on a community level. The relationships among these are presented in Figure 2 and described below in relation to findings from the empirical data. In the final section of the paper, we discuss how this is relevant in relation to a sense of collective coherence. As there is only limited literature using a salutogenic perspective in a Sámi context [33,34,35], we also made an effort to distinguish data that might question the theory’s applicability or relevance in this context.

## 4. Findings

The findings are presented in relation to the illustration in Figure 2. GRRs, at the positive end of a continuum, are shown on the upper half of Figure 2, and conceptualized in relation to the Sámi community as Social Organization; Familiarity with EoL Care, Collective Cultural Heritage; Expression of Spirituality; Support from Majority Care Systems; and Brokerage, with related factors found in the data, under each heading; note that these codes are not mutually exclusive but presented separately for the sake of clarity. These positive features support the key components of a sense of community coherence, i.e., Comprehensibility, Meaningfulness and Manageability. The lower left side of the figure shows the opposite, negative end of the GRR-GRD continuum, with the relatively few features that work to diminish a sense of community coherence that were found in these data. 

All individuals cited below are Sámi, if not otherwise noted. 

### 4.1. Generalized Resistance Resources

#### 4.1.1. Social Organization

The most central organizational form in these data relating to EoL is the extended family, a feature of Sámi life that is self-evident and taken for granted, as this woman says, “*Family is just there. It’s just there.”* (FGD 4) There were also many stories about its’ importance; one woman working as a home health aide, said: 

In some way I feel like I think we have stronger ties to each other in some strange way. They [non-Sámi] say, usually say like “*yes, you Sámi, you’re so attached to your family […] I think that…at times of death…it gives us strength when there is serious disease. And it’s something, I think, that we should boast about…*” (FGD 4)

This comment exemplifies a number of important resources, i.e., social support, commitment, and cohesion to one’s cultural roots, and suggests others, i.e., cultural stability and coping strategies, with a sense of pride and happiness apparent in her voice. 

Cultural stability is also demonstrated through traditions that are incorporated into an existing and functioning extended family system, which becomes particularly evident in EoL situations. A lawyer speaks of his own role in his extended family, but also refers to the informal *Verdde* (North Sámi spelling) system, for reciprocity between reindeer-herding Sámi families and non-reindeer-herding families, both Sámi and non-Sámi [36]. *Verdde* involves the mourning family being helped and supported with practical aspects of life, particularly around the time of burial:

*I’m participating here [at the churchdays] because I think I have…as a member of this clan I have experienced lots of…I have been part of this family system, relation…this care in the end-of-life. There are some...lots of things to think about. Lots of traditions in my area and also in the other Sámi areas. And also dealing with this…subject in my profession.*

(FGD 3)

#### 4.1.2. Familiarity with End-Of-Life Care

The importance of self-determination with the ability to steer resources—physical, practical and psychosocial—appeared often to be related to having knowledge and experience oneself or access to it through others that were trusted. This is evident in a conversation between two women, a nursing assistant (D6) and a Norwegian church functionary (D2, non-Sámi) about in-patient care for D6′s dying relative:

D6: *I felt like my workmates were a support during that time.*

D2: *But then you were steering it a bit when you decided what you would need…it was you who was the closest as well, and so it was you who pressed the button when they [staff] should come in.*

D6: *yes, but I…but you know what it was? I’m both an assistant nurse and a relative, and then I could sometimes feel ‘what is what’? But I didn’t feel badly about it. I had to call you know, when I felt like I couldn’t manage, then I called and so another workmate came in.*

(FGD 4)

This situation illustrates a positive collaboration between formal care provision and family, simplified by an overlap with a family member with professional competence and familiarity, not only with care but also with the care providers. However, this was not always the case, and perhaps the most striking GRDs were those describing the negative effects of lacking such familiarity. The following quote illustrates a situation in which a woman describes her sense of being lost in an unfamiliar care system, which also defined the role of family differently than was the norm in her community:

*Because we were very lost, both mama and me, in the health care system. And these words that are so important, care-planning and …if you…you know what you can get and not get from the health care system when you are in…you know, death’s waiting room, that was very confusing for us. And as Sámi, we are very close to our extended family and it is, it was very confusing because we’re used to doing everything ourselves and then suddenly the health care system tells me “you shouldn’t be the care provider, you should be family, you should brighten up her last phase of life”. And it was really hard to figure out where the line should be drawn. And I realized afterwards that I maybe should have asked for help earlier. As an only child and as a Sámi, it was very…And finally Mama said stop, she said “we reached the limit, it’s no longer dignified for you, as my child, to care for me” And that feeling of being lost in the health care system as a Sámi, in death’s waiting room, I feel that so strongly, that a little book or something is needed.*

(FGD 4)

This woman is unusually clear about a need for guidance for her, as a Sámi, in dealing with EoL issues. This unfamiliar situation demanded a departure from that which she was used to and which was tradition for her. She expresses a clear sense of vulnerability, for both her mother and herself, in as she says “*… death’s waiting room”.* (FGD 4)

#### 4.1.3. Collective Cultural Heritage

While the description above relates to problems arising as Sámi norms clash with the majority health care system, many stories instead spoke to the importance of and pride in Sámi traditions and cultural features such as food, nature, and reindeer-herding as providing both structure and support at the EoL. A woman working for a municipality told a story of noting the importance of familiar sensory experiences during her visit to a residential care home for the elderly, relating it to information from a local government survey of older Sámi:

*And I pushed that ’skåerrie’ [a piece of treated reindeer skin with a recognizable scent] across the table to the two ladies sitting there. And they just went ’mmm, skåerrie, ohhhhh’. So you can see how much it means, things you may think are trivial, but they are memories and they are childhood and they are family and they are just the culture, they are so much…*

*There were three things everyone wanted [refers to the survey]. And these were that when they got older and moved to a care home, they wanted to still have their language with staff that could speak Sámi, and Sámi food, and they wanted to be able to be with other Sámi.*

(from data collection 2, go-along group discussions)

Sámi languages were mentioned repeatedly in discussions and stories, as both an important aspect of care, as well as a means to deal with grief. A non-Sámi priest, experienced in working with Sámi congregations, exemplifies this, saying:

*And you know, it’s not possible to carry those feelings across in a second or third language, and so therefore we need to use our mother tongue in such times, that is at a death bed or at the funeral […] as an important part of the process, the healing process or the grieving process*.

(FGD 1)

Another, less frequently described and, as observed below, often silenced, phenomenon, that of using traditional Sámi medicines, was spoken of by a young woman who had her own experience of life-threatening disease:

*And then there’s a lot, you might say alternative medicine, that is not drugs, but how you earlier maybe drank chaga-birch tea and blueberry, that is these things that are around, that have been used for a long time. So this knowledge exists. But it’s very very…hush hush and secretive.*

(From data collection 3, ACP discussions)

As she continues, the intertwined relationship between traditional treatments and spirituality also becomes evident:

*They [the family] called ’govhlar’, what’s it called, kind of healers, shamans, who you believe in, in the Sámi culture and who still remain. And you call them and ask them for help so that they can heal and make it take a turn for the better now […] No one has visited me in person, but it’s about these strong forces that work through the mind.*

(From data collection 3, ACP discussions)

#### 4.1.4. Expressions of Spirituality

As observed in the above quote from the young Sámi woman, spirituality was evidenced in many aspects of life, and not only isolated in formal religious rituals. One Sámi-specific means of dealing with feelings such as grief, but also a means of making death both comprehensible and meaningful, is *yoik*, a traditional Sámi a cappella song. Yoik is both a verb and a noun, and one yoiks not to someone or something, one becomes what one yoiks [37]. This man speaks of how he commemorates his connection to his ancestors through “person yoik”: “*It is very important when it’s related to death. Just to be able to go on and remember or spend time with someone through their person yoik.”* (FGD 1)

Yoik is also a means for communicating joy. A social worker tells about her sister’s funeral and its’ effect on others less familiar with Sámi traditions:

*But during the funeral there was yoik and everything. And then these people, her workmates, she said it was fantastic to be at a Sámi funeral. We’re there with koltar* [traditional clothing in the national colors of red, blue, yellow and green] *and all, it’s not all in black and so, but it was, yes it was so very nice.*

(FGD 4)

In these data, expressions of spirituality often reflected a way of life. Based on her experience of EoL situations, this non-Sámi woman who had worked with Sámi churches for many years described how animals could be a form of protection, as well as support:

*I’m thinking about if I experienced anything […] the animals had such …importance […]. And everyone had their own animal that watched over them and that they were preoccupied with, and then I thought, that this is a Sámi thing. Understanding animals and that way of being observant, it comes from their way of living…*

(FGD 2)

However, Christianity was dominant in the descriptions of formal religion at the EoL, with many variations between individuals and families, but as a self-evident part of many Sámi’s identities. As a woman said: “*We are so influenced by it* [Christianity] *and we’re Christian of course, we are more Christian than Swedish in general.”* (FGD 4)

Another aspect of our data we categorize as relating to spirituality regards an immaterial legacy of Sámi values and norms. Many ways of dealing with responsibilities in EoL situations were spoken of, and this male, non-Sámi church parson speaks of dangers in neglecting such responsibilities:

*even though you are related […] there are these moral obligations. There is also a…it’s also dangerous to say no […], yes, it is unheard of, so to speak. You can also bring misfortune over yourself then […] since it is such a very strict norm*

(FGD 3)

Sámi norms thus demand both engagement and continuity with one’s cultural roots, with many speaking of responsibilities at the EoL as indisputable. While Sámi people are often depicted in stereotypes, i.e., “*strong and solitary”*, “*uncomplaining”*, and “*quiet and self-sufficient”* (FGD 4) we also find descriptions that challenge these clichés, for example as told by this woman from a remote area:

*I’ve grown up with X siblings, and if one is sick, we are all sick and everyone wants to contribute […] Father always said ‘it’s important to show your feelings’. And we have cried together and I have a sibling who died in an accident […] I thought when my brother died […] then that it was punishment from God. But then my grandmother said ‘it’s not punishment from God, but we have different trials, because life is a school that can be hard sometimes, and maybe his time had come’. And that has given me strength in my grief process, thinking that […] his time had come. […] it’s a trial but not a punishment. There’s no shame. No sense of shame.*

(FGD 4)

However, shame and silence are spoken about by several participants as a way of dealing with sickness and death. A woman speaks of this in relation to her own experiences of caring for a close family member at the EoL:

*Yes, and in the Sámi world, there is a zipper here. You shouldn’t talk about feelings, you shouldn’t…you feel shame if you are sick because then there is something evil you have…sort of…You have maybe…it is a punishment from God. And there is a lot of shame connected to talking about it and opening yourself up.*

(FGD 4)

Despite these traditional norms, several participants, as observed above, expressed a desire for more openness. As the woman quoted directly above continues: “*But I also have to say thank you for people daring to talk…It feels so very…very beneficial”.* (FGD 4)

#### 4.1.5. Support from Majority Care Systems

In the text above, there are illustrations of problems arising at the intercept of the majority health care system and the Sámi community. However, there were also descriptions of well-functioning support. One woman, living in a remote area, describes how she, along with her sisters, struggled to move their mother from inpatient care to her home during the last days of her life. While there was the conflict with the hospital, she described the support they felt from the primary health care system:

*If you only knew how stubborn we had to be. Everyone came and said, ‘do you really understand what kind of responsibility you are taking on, the responsibility you have?’ But then I call the community nurse [mentioning her by first name] and so I said…I told about how it was and that we were thinking of bringing Mama home, but that they advised us not to. “Take [mentions mother by name] home, I’ll come and help you” she just said. So my sister went to her [the community nurse] and brought back a load of things to help…*

(from data collection 2, go-along group discussions)

Help was also provided by representatives of voluntary organizations, described as enthusiasts, working with municipalities. In the following quote, ongoing work in an area in which government agencies appear to take their mandate to support Sámi culture seriously is referred to, here in relation to residential care homes for the elderly. A woman who works with traditional handcrafts says:

*and there are always some fantastic enthusiasts who work for the Sámi population, the elders, who should be able to have a place in residential care homes, on special units where they feel at home, where staff who applies for those positions, they are able to study Sámi language to be able to meet their needs […]. And then you need some enthusiasts who, along with their maternal and paternal grandparents, get a foot in and have the strength to get the municipalities to… so that this unit…it should be reserved for…it should just be for Sámi.*

(FGD 2)

While in the above quotes, participants describe actual help received from majority social care systems in collaboration with Sámi support systems, a woman who had worked in home health care speaks of her visions for the future, pointing to a sense of security and cultural context in daily life as crucial:

*...for us Sámi, our own TV and group room, so we can see Ođđasat* [Sámi news program] *without the laedtieh* [non-Sámi] *who question ’what’s that you’re watching?’…no, I say, we should have our own* […], *we can be in the same unit* […] *but then when we should have our food and such, then we can go and eat in their dining room. But we should have our own…it should be the kind of cooking that we cook, so we can make bovtsen bearkoe* [reindeer meat] *and we can guelie voessjedh jih maelie laejpie bissedh jih numhtie* [cook fish and fry blood bread and all that]…

(FGD 2)

#### 4.1.6. Brokerage as a Bridge between Cultures

The ability to deal with EoL care is strongly supported by the extended family system, which activates a wide range of resources in a broad network. In our data, we found frequent mention of how a variety of people with formal education and/or experience who were within the reach of the extended family, were used as resources, able to fill different roles. We found examples of people acting as advocates for the ill person and family, assistant nurses, community nurses, deacon/deaconess, home help aides, hospital clergy, hospital translators, physicians, priests, registered nurses, and representatives of volunteer organizations. Most notable is that these people act as a bridge, i.e., as brokers, between the Sámi community and resources and knowledge about EoL from the majority society. 

In the preceding text, we provided one example showing how a Sámi woman negotiated her double role as a family member and an assistant nurse and also described how the extended family and Sámi culture are an organizational form resting on both a strong sense of responsibility and sharing such responsibility in particular manners. Another participant spoke about how EoL home care for his Sámi mother was made possible through the help of a relative he describes as “*a woman who had sure-fire competence and was able to take the bulk of responsibility*.” (FGD 3)

A woman reflects on the benefit her father had in having a Sámi-speaking community nurse during his last phase of life: *“I know my father had a community nurse who spoke Sámi, and she was the one he had most trust in…it was her that he…he waited for her to come, so he could talk.”* (from data collection 1, individual interviews)

A non-Sámi priest describes the effect of a psalm the priest sung in a familiar language in the last days of a Sámi man’s life:

*And then I sang, and so suddenly, the nurse, she happened to be there and suddenly he [the dying Sámi man] lifted one of his hands and so he began…you could even hear it, how he started to hum along, and then she [the nurse] said ‘that’s never happened before’, she said. Yes, but now it happened. It touched something inside him…*

(from data collection 1, individual interviews)

The Sámi lawyer summarizes well the importance of the broker role, bridging different systems and types of knowledge needed to deal with EoL issues among Sámi communities:

*And most people don’t have the energy and the knowledge that they have [in the formal health care system]. So that’s why it’s so important that someone in the church and in the health care system knows what’s important to Sámi, and asks about it, isn’t it? And it is…that we know too little, really both the church and health care and all those places. That’s why it’s so important, what you see here [in this research].*

(FGD 3)

### 4.2. Generalized Resistance Deficits

We have already mentioned most of the GRDs found in our data in the above text, contrasting them with the positive qualities of GRRs, for example, lack of communication in one’s own language, as well as a sense of disorientation or lack of familiarity with, or at its’ most extreme, conflicts with health and social care systems. In addition, one other GRD found in our data concerns the lack of an extended family. This is evident in this comment by a priest:

*I was thinking, horrors of horrors is one of…if there is a Sámi who doesn’t have a strong family who can stop, then you are …abandoned to a system that has it’s bureaus and doesn’t have time or space or possibility or the desire to let the family go in and be involved. And the people of the church even have [refers to internal lack of understanding for Sámi culture and needs even within the church]. Because we wanted the family to ride with the casket but ‘no that’s not what we usually do’…*

(FGD 3)

The priest ended his comment by pointing out that even with strong family ties, conflicts can occur not only with the health or care systems but also with the church when norms and traditions collide. 

## 5. Discussion

In this study, we see a new perspective of the extended family in Sámi EoL contexts as including a broad network with far-reaching arms. This family/network functions as a social organizational system and form of support in which those involved have clear roles and responsibilities. Through this network, a wide range of GRRs are accessed and various resources can be activated when needed. This extended system plays a central role in linking both what is often called an “informal” community-based care system with the formal care provided by the majority society, in best cases appearing to support EoL care becoming meaningful, comprehensible, and manageable.

Despite some potential limitations in our data, e.g., the selective nature of our three samples with most informants self-defined Sámi from Northern Sweden, and a substantial portion of data deriving from Sámi church Days, we still find that many of the GRRs we see in our data that are accessed through extensive network systems are well in line with those described by Horsburgh and Ferguson [38]. To use their terms, we see GRRs related to material resources; knowledge and intelligence (e.g., knowing the world and acquiring skills); coping strategies rooted in tradition; social support, commitment, and cohesion with one’s cultural roots; cultural stability; rituals; religion; and philosophy. In combination, these comprise a world view and way of life which seems to often provide a “stable set of answers to life’s perplexities” [39], and also includes relationships with nature and animals. While Horsburgh and Ferguson [38] also name personal attributes, those are beyond the scope of this study, as we focus on a sense of community coherence here. 

However, as observed previously, the characteristics of a sense of community coherence are collective versions of components important on an individual level [32]. We found only limited prior research based on Antonovsky’s work in Sámi populations, with all focusing on the individual level using the SOC instrument [33,34,35]. Abramsson et al. [33] found that while there were relatively few differences between the majority Swedish sample and the Sámi sample in their study, Sámi working with the traditional occupation of reindeer-herding were found to have lower SOC, particularly in regard to comprehensibility, but had notably high levels of meaningfulness. Hassler and Eklund [34] compared self-reported health with results from the short version of the SOC instrument, finding that the studied Sámi had low levels of mental health and experiences of structural discrimination similar to another official minority group in Sweden, the Roma people. Alex [35] studied resilience among old Sámi women. The Sámi women’s narratives generally described a high degree of resilience; discrimination was described as negatively affecting their resilience. This leads to several questions, previously raised by Tishelman [40], e.g., how can the same manner of instrumentalizing SOC, as in the existing questionnaire, be both supra-cultural and gender-neutral, particularly as it aims to investigate “a succinct formulation of a world view, of the web of linkages between the person and his or her world” [41]. We note the importance of this linkage clearly in our data. While our data may be considered limited in that we apply no measurement of SOC on the individual level, through analysis of our qualitative data and consideration of the components of community coherence, we have instead provided a variety of examples leading to a new understanding of what supports the components of community SOC. We find that comprehensibility is heightened by a clear understanding of both roles and responsibilities, as well as norms and values that have permeated the Sámi culture across generations. This knowledge, and its dissemination through processes of enculturation [1,2] seem to help make EoL care both meaningful, comprehensible and manageable. Meaningfulness is indeed one of the most prominent aspects of these data, and difficult to disentangle from comprehensibility and manageability, as both concrete and general descriptions could be permeated with pride and joy in Sámi traditions and culture. What is striking in these data, is the importance of familiarity with and respect for this from professionals. Manageability is also heightened by familiarity, which means that contacts with the majority health care system may involve potential risks to the sense of community coherence, if cultural values, traditions and norms are not respected.

In these data we found a variety of people who acted as cultural brokers, seeming to help negotiate risks, access resources in both material and immaterial forms, and avoid distress. These individuals appear to have a key role in mediating contact between the majority health and care systems, and those based in the Sámi community. In these data, cultural brokers were trusted people with knowledge about how the majority care system worked and familiarity and/or sensitivity about the Sámi context. These people were often but not always Sámi themselves, as even others, e.g., non-Sámi community care nurses, could act to bridge cultures. We have however found few descriptions of cultural brokers specifically for EoL care. One exception is Aboriginal or Indigenous Liaison Officers who work in various ways to support culturally appropriate EoL care [17].

One role of such cultural brokers can be seen as promoting cultural safety. Mehus et al. [42] found that bilingual health care staff in Norway were able to use their competency in Western medicine and familiarity with the majority health care system to provide culturally safe care for the Sámi population, illustrating a form of cultural brokerage. Other examples of cultural brokerage for Sámi in Norway through use of Sámi staff are illustrated by Andreassen Devik and Olsen 2020 [43] based on municipal care for elderly Sámi, and Larsen [13], as who studied relatives to Sámi patients with dementia.

Mehus et al. [42] mean that in order to achieve cultural safety on a group level, all health professionals who meet Sámi patients and families should reflect on the extent to which cultural competence and sensitivity are present in their workplaces. Andreassen Devik and Olsen [43] conclude that cultural sensitivity is even a prerequisite for person-centered care for Sámi. While the term cultural sensitivity is most often used in English-language literature, its use is not always consistent [44], and in our prior work, we have used the term cultural humility [45] instead. Whichever term is used, we argue based on this study, that most important is that these qualities lead to cultural safety for Sámi or other Indigenous groups in contact with majority health and social care systems. As Schill and Caxaj [46] highlight from their review of palliative care in rural Indigenous settings in several countries, cultural competency may lead to a framework of cultural safety but is not sufficient in itself. They also emphasize that strategies leading to cultural safety contribute to decolonization of care, by heightening awareness of colonialism, racism and discrimination, and thus supporting partnership building and sharing of power and decision-making in care situations. 

## 6. Conclusions

In conclusion, according to Antonovsky, a prerequisite for discussing SOC on a community level is the existence of an “identifiable collectivity”, with sense of shared identity [30], pointing out that a group SOC can be a characteristic of an oppressed or self-aware minority. The insight achieved through this study suggests that there is a robust basis among self-defined Sámi for well-functioning EoL care; a challenge is in developing supportive interactions with the majority health and social care systems that further support and complement these structures, for partnership in developing care that is meaningful, comprehensible and manageable even in potentially difficult EoL situations.

## Figures and Tables

**Table 1:** Overview of previous data collections.

	Individual Interviews ^1^	Go-Along Focusgroup Discussions ^2^	Individual Interviews ^3^
Age (year)	25–83	19–78	26–84
Participants, number (*n*)	15	12	31
Women (*n*)	8	7	18
Men (*n*)	7	5	13

See references. ^1^ [1] ^2^ [2] ^3^ [3].

## Data Availability

For original data, please contact the corresponding author; ethical approval does not cover making data openly accessible.
